# Non-Invasive Sampling Reveals Colony-Scale Spatial Heterogeneity in Cadmium Concentrations in Atlantic Puffin *Fratercula arctica* Droppings in Iceland

**DOI:** 10.3390/ani16172689

**Published:** 2026-08-28

**Authors:** Jakub Skorupski, Arkadiusz Nędzarek, Remigiusz Panicz, Piotr Eljasik, Natalia Śmietana, Erpur Snær Hansen

**Affiliations:** 1 Institute of Marine and Environmental Sciences, University of Szczecin, Mickiewicza 16 St., 70-383 Szczecin, Poland; 2 The Centre for Molecular Biology and Biotechnology, University of Szczecin, Wąska 13 St., 70-415 Szczecin, Poland; 3 Faculty of Food Sciences and Fisheries, West Pomeranian University of Technology in Szczecin, Kazimierza Królewicza 4 St., 71-550 Szczecin, Poland; 4 South Iceland Nature Research Centre, Ægisgata 2, 900 Vestmannaeyjar, Iceland

**Keywords:** Atlantic puffin, biomonitoring, cadmium, central-place foraging, droppings, ecotoxicology, seabirds, trace metals

## Abstract

Cadmium (Cd) concentrations in Atlantic puffin *Fratercula arctica* droppings were assessed during chick rearing using non-invasive sampling at nine Icelandic colonies. One fresh dropping was collected immediately after directly observed defecation from each of 159 different individuals in June–July 2018, and Cd was quantified by graphite furnace atomic absorption spectrometry on a dry-weight basis. Reported Cd concentrations were strongly right-skewed (median, 155.2 µg kg^−1^ dry weight; range, 1.1–3539.2 µg kg^−1^) and differed among colonies in the primary unadjusted comparison (Kruskal–Wallis, *p* < 0.001), with Holm-adjusted Dunn tests identifying 9 of 36 significant colony pairs. In a secondary model including dry sample mass, the colony effect remained strong (Wald χ^2^(8) = 108.75, *p* < 0.001), and adjusted geometric means spanned 24.2–506.9 µg kg^−1^ dry weight (20.9-fold). Variance partitioning yielded an ICC of 0.261 (95% confidence interval, 0.104–0.519), which is interpreted as a model-dependent estimate given the nine colonies. No association with chick-provisioning prey composition or breeding performance remained detectable after false-discovery-rate correction. The 2018 dataset therefore shows pronounced colony-scale contrasts in reported Cd concentrations in droppings. Mechanistic attribution requires direct prey measurements and multi-year replication.

## Simple Summary

Seabirds can provide information about contaminants in marine food webs, but conventional tissue sampling often requires capturing or handling birds. We measured cadmium in 159 fresh droppings, one from each of 159 different Atlantic puffins, at nine breeding colonies in Iceland. Mass-adjusted model estimates differed approximately 21-fold among colonies after accounting for dry sample mass. About one-quarter of the total variation occurred between colonies, whereas most variation occurred among birds within the same colony. Chick-provisioning prey composition estimated from prey-load photographs and breeding performance did not explain these differences after correction for multiple statistical tests. The results show that non-invasive sampling of droppings can identify spatial differences in cadmium concentrations that are consistent with possible differences in recent dietary exposure, without capturing or handling birds. However, adequate numbers of birds must be sampled within each colony because concentrations vary substantially among individuals. Repeated sampling and direct measurements of cadmium in prey are needed to determine the sources, persistence, and ecological significance of the observed differences.

## 1. Introduction

Marine birds have long been used as sentinels of environmental change because they integrate ecosystem processes across trophic levels and spatial scales that are difficult to sample directly [1,2]. Related biomonitoring approaches have also been applied in Iceland using other sentinel vertebrates [3]. For chemical contaminants, a principal challenge is obtaining information at ecologically meaningful resolution while minimizing disturbance and avoiding destructive sampling. Non-invasive matrices can address this constraint, but their interpretation requires an explicit understanding of the time window and exposure pathway represented and of potential methodological biases [4].

Cadmium is a priority toxic metal of global concern, released through both natural processes and anthropogenic activities and characterized by persistence and the capacity for bioaccumulation in food webs [5]. Although cadmium toxicity has been most extensively characterized in mammals and human health contexts, exposure in wildlife is generally treated as undesirable, particularly in multi-stressor environments [5,6,7,8]. In marine systems, seabirds can therefore provide a biologically relevant signal of cadmium availability in prey fields, especially when sampling is aligned with known foraging constraints during breeding [1,2].

Bird droppings, which may include both fecal and urate components, have been proposed as attractive matrices for contaminant biomonitoring because they can be collected opportunistically and repeatedly without capturing birds and may reflect recent dietary intake rather than long-term body burdens [9,10]. At the same time, the inferential relationship between concentrations in droppings and internal accumulation is not universal and can vary by element, exposure level, physiology, and matrix-specific processes. Consequently, rigorous analytical quality assurance and quality control and transparent treatment of values near detection or quantification limits are essential [4,10]. When these constraints are addressed, monitoring based on droppings can complement more established matrices such as feathers, which integrate exposure over longer, molt-dependent time windows and may obscure short-term spatial contrasts relevant to breeding ecology [11].

The Atlantic puffin *Fratercula arctica* is a central-place forager during chick rearing, and colony-level performance is sensitive to prey availability and foraging conditions, making the species an informative model for connecting exposure signals to ecological drivers [12,13]. Diet composition in Atlantic puffins is routinely inferred from bill-load observations and photographs, and multi-method comparisons have emphasized that each dietary proxy reflects a specific component of foraging ecology and sampling bias [14]. In Iceland, background metal burdens in puffins have recently been characterized using feathers from multiple colonies, providing an important benchmark but leaving unresolved whether cadmium exposure during breeding shows finer colony-scale heterogeneity consistent with short-term foraging ecology [11]. Furthermore, Icelandic environments are influenced by strong atmospheric and coastal transport processes, including dust mobilization and deposition, which may contribute to spatial structuring of trace-element availability [15].

The present study assessed whether Cd concentrations in droppings during chick rearing exhibited colony-scale spatial heterogeneity across Iceland and whether this heterogeneity was statistically associated with colony-level ecological variables. The study extends previous tissue- and feather-based biomonitoring by assessing Cd concentrations in puffin droppings across multiple Icelandic breeding colonies. Using non-invasively collected fresh droppings from nine colonies, three hypotheses were evaluated: (H1) Cd concentrations in droppings differ significantly among colonies; (H2) mass-adjusted colony Cd concentrations in droppings covary with colony-level chick-provisioning prey composition; and (H3) higher colony-level Cd concentrations in droppings are associated with reduced breeding performance. Analyses under H2 and H3 were treated as exploratory because mechanistic attribution was constrained by the limited number of colonies and the absence of direct Cd measurements in prey or environmental matrices.

## 2. Materials and Methods

### 2.1. Study Area and Field Sampling

**Figure 1:**
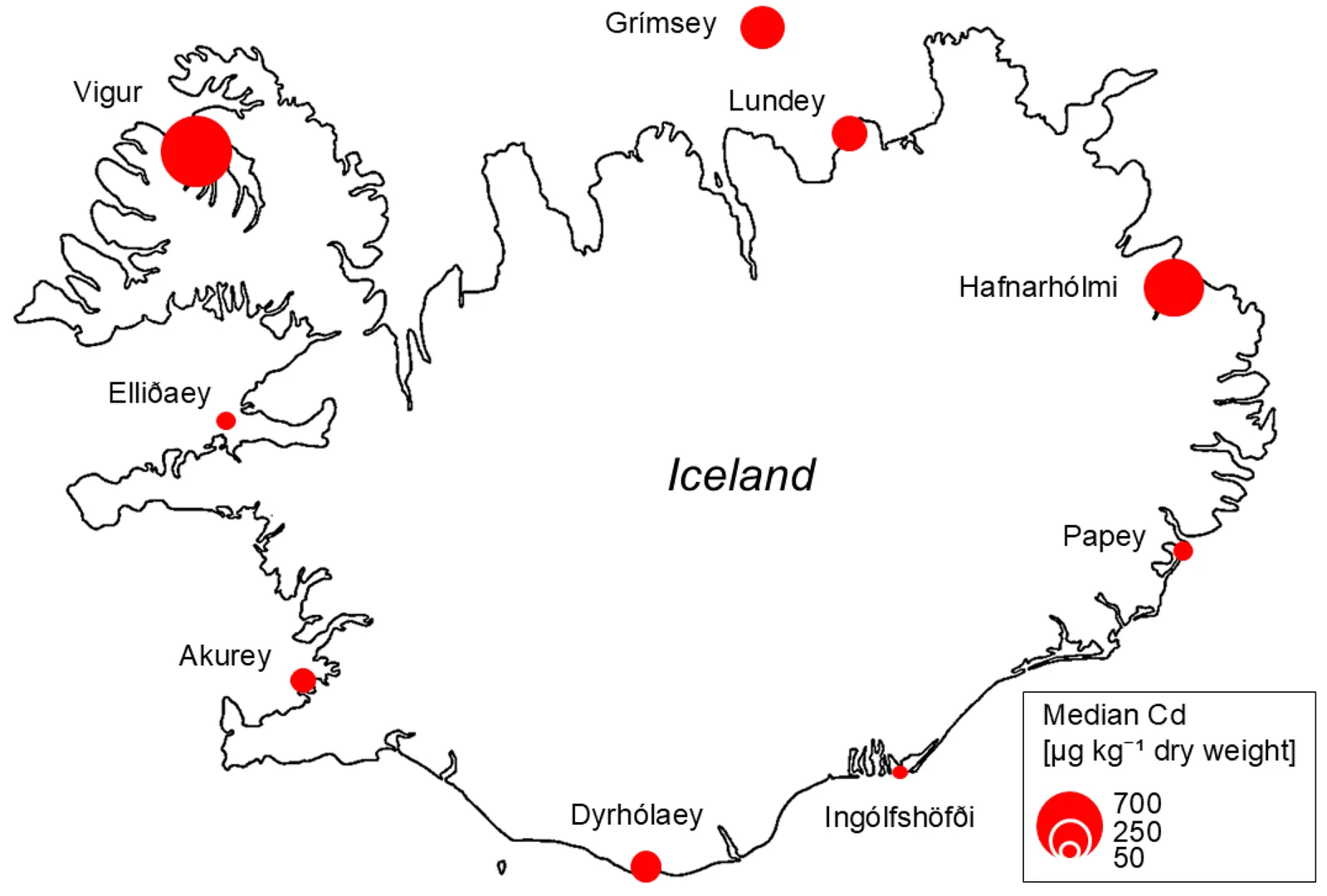
Spatial distribution of colony-level median cadmium concentrations in fresh droppings from Atlantic puffins *Fratercula arctica* across Iceland, June–July 2018. Circle area is proportional to colony median Cd concentration (µg kg^−1^ dry weight). Exact colony coordinates are provided in Section 2.1. The map is intended for site location rather than distance measurement.

Fresh droppings (*n* = 159) were collected non-invasively during the Atlantic puffin breeding season (June–July 2018) within nesting areas across nine colonies in Iceland, spanning contrasting coastal settings (Figure 1, Table S1). Sheltered nearshore islets within bays and fjords were represented by Akurey in Kollafjörður (Faxaflói) (64°10′23″ N, 21°57′58″ W; *n* = 27), Elliðaey in Breiðafjörður (65°08′35″ N, 22°48′56″ W; *n* = 14), Hafnarhólmi at Borgarfjörður Eystri (65°32′34″ N, 13°45′24″ W; *n* = 16), and Lundey in Skjálfandi Bay (66°06′50″ N, 17°22′12″ W; *n* = 15). More exposed offshore islands in fjord systems were represented by Vigur in Ísafjarðardjúp (66°03′22″ N, 22°49′46″ W; *n* = 19), Papey off the Eastfjords (64°35′20″ N, 14°10′15″ W; *n* = 14), and Grímsey offshore on the north coast (66°32′47″ N, 17°59′36″ W; *n* = 21). Mainland headlands with seabird cliffs were represented by Dyrhólaey (63°24′13″ N, 19°07′05″ W; *n* = 16) and Ingólfshöfði (63°47′59″ N, 16°38′21″ W; *n* = 17). Each sample represented one fresh dropping collected immediately after a directly observed defecation event. The focal bird was observed until collection, and no individual was sampled more than once. Thus, the 159 samples represent 159 different individuals. Sampling personnel wore disposable laboratory gloves, and each dropping was collected with a single-use sterile spatula into a sterile plastic container. Samples were frozen immediately after collection and transported at −30 °C. Operationally, a dropping was classified as fresh only when defecation was directly observed and collection followed immediately.

Field sampling was conducted within Iceland’s national Atlantic puffin monitoring program coordinated by the South Iceland Nature Research Centre (Náttúrustofa Suðurlands), and the 2018 monitoring is documented in its progress report to the Environment Agency of Iceland [16]. Breeding performance data were obtained from Iceland’s national puffin monitoring program [16]. Colonies were visited twice, in June and July 2018. During each visit, the same set of pre-marked burrows was inspected using burrow cameras with built-in infrared illumination. The number of burrows included in the analyses varied among colonies, ranging from 35 to 62 (Table S2). Four monitoring indices were derived: burrow occupancy (BO; proportion of monitored burrows containing an egg), hatching success (HS; proportion of eggs that hatched), nesting success (NS; chicks assumed to fledge per egg laid), and productivity (P; the product of BO and NS, representing chicks assumed to fledge per monitored burrow), together with their associated standard errors. Large chicks observed late in the nestling season were assumed to fledge successfully. For the national monitoring program, the visit schedule had been revised from 2017 onward: the June survey was conducted approximately 10 days earlier than in 2010–2016, and the colony visit order in both June and July was adjusted to make visit timing within the breeding season as comparable as possible among colonies [16].

Chick-provisioning prey composition was estimated at the colony level from photographs of adults carrying prey to chicks, with prey items classified as lesser sandeel *Ammodytes marinus*, Atlantic herring *Clupea harengus*, fish larvae, or “other” prey [16]. These prey-load photographs characterize chick provisioning and were not treated as a measure of the complete adult diet of the birds producing the droppings. Diet results were analyzed as prey-category proportions per colony, with sample sizes indicated by the number of prey-load photographs analyzed.

### 2.2. Sample Processing and Cadmium Determination

Samples were dried at 60 °C for 24–48 h and weighed after drying. Dry mass was used to express concentrations on a dry-weight basis.

Digestion was performed by high-pressure microwave mineralization using a Speedwave Xpert system (Berghof Products + Instruments GmbH, Eningen, Germany). An aliquot of dried material was digested in 2.0 mL of ultrapure concentrated nitric acid (HNO_3_; Merck KGaA, Darmstadt, Germany). The digestion program comprised an increase to 170 °C over 5 min followed by 5 min at 170 °C and 30 bar, then an increase to 200 °C over 3 min followed by 20 min at 200 °C and 30 bar, and a final 10-min step at 50 °C and 25 bar. After cooling to room temperature, digests were brought to a final volume of 5 mL with Milli-Q water (18.2 MΩ·cm; Merck KGaA, Darmstadt, Germany).

Cadmium concentrations were determined by graphite furnace atomic absorption spectrometry using a Hitachi ZA3000 Series Polarized Zeeman Atomic Absorption Spectrophotometer (Hitachi High-Technologies Corporation, Tokyo, Japan) equipped with a Hitachi PyroTube CII HR graphite tube. Operating conditions were: 228.8 nm wavelength, 7.5 mA lamp current, 1.3 nm slit width, background correction, PMT voltage 310 V, argon 6.0 carrier gas, and 20 µL injection volume. A 1000 ppm Pd(NO_3_)_2_ solution (SIGMATIK, Wrocław, Poland) was used as the matrix modifier, with a modifier injection volume of 5 µL. Two injections were made per digest. The furnace program was: drying at 80–140 °C for 40 s (200 mL min^−1^ gas flow), ashing at 300 °C for 20 s (200 mL min^−1^), atomization at 1500 °C for 5 s (30 mL min^−1^), cleaning at 1800 °C for 4 s (200 mL min^−1^), and cooling for 10 s. Concentrations are reported as µg kg^−1^ dry weight.

### 2.3. Quality Assurance and Analytical Performance

Method performance was assessed using the certified reference material ERM^®^-BB422 (European Reference Materials, European Commission, Joint Research Centre, Geel, Belgium), for which the certified Cd mass fraction is 0.0075 ± 0.0018 mg kg^−1^ dry mass. Cd recovery in the analytical series was 98%. Calibration covered 0–10 µg L^−1^ (0, 2, 4, 6, 8, and 10 µg L^−1^), with three measurements at each level, linear regression accepted at R^2^ > 0.995, and control-standard recovery accepted at 90–110%. Quality-control checks were performed approximately every 10 samples. Routine laboratory acceptance criteria for replicate instrumental injections were relative percent difference (RPD) ≤30% near the LOQ, ≤20% at 3–10 × LOQ, and ≤10% well above the LOQ. Glassware was cleaned in 1% HNO_3_ and rinsed with ultrapure water. After high-Cd samples, the autosampler needle was automatically rinsed and the graphite tube was cleaned by the furnace cleaning step to minimize carryover.

Instrumental sensitivity during the analytical campaign was evaluated in the final solution, with solution-phase LOD and LOQ values of 0.2 and 0.67 µg L^−1^, respectively. These values do not, by themselves, define sample-specific method limits on a dry-weight basis, because such limits depend on the amount of dry material represented in each digest and the sample-specific analytical calculation.

### 2.4. Statistical Analyses

All analyses were performed in Python 3.11.2 (Python Software Foundation, Wilmington, DE, USA) using NumPy 1.24.0 [17], pandas 2.2.3 [18], SciPy 1.14.1 [19], and statsmodels 0.14.3 [20]. Dunn’s post hoc tests [21] and Moran’s I permutation tests [22,23] were implemented in custom Python code, with multiplicity controlled using Holm [24] or Benjamini–Hochberg false discovery rate adjustments [25] via statsmodels 0.14.3 [20]. Figures were produced in matplotlib 3.7.5 [26].

Statistical significance was assessed at α = 0.05 using two-sided tests. Where multiple testing was performed, Holm-adjusted *p* values for Dunn post hoc tests or Benjamini–Hochberg false discovery rate q values for colony-level screening correlations were used.

#### 2.4.1. Data Preparation and Unadjusted Among-Colony Comparisons

All statistical analyses were conducted using dry-mass-standardized Cd concentrations. Distributional properties of Cd concentrations and dry sample mass were assessed using the Shapiro–Wilk test [27]. Given the strong skewness, descriptive summaries were based on medians and interquartile ranges, and inferential analyses used rank-based tests and log-transformed modeling, as specified below.

Among-colony heterogeneity in unadjusted Cd concentrations was tested using the Kruskal–Wallis test [28]. Where a significant omnibus effect was detected, pairwise post hoc comparisons were conducted using Dunn’s test [21], with Holm adjustment to control the family-wise error rate across all colony pairs [24]. Because sample-specific analytical limits cannot be reconstructed from the retained records, low observations were not censored post hoc. Instead, robustness of the primary colony comparison to the lower tail was assessed by repeating the Kruskal–Wallis test after excluding reported Cd concentrations < 11.6 and <38.8 µg kg^−1^. These cut-offs correspond to dry-weight equivalents of the solution-phase LOD and LOQ calculated using the largest reported dry sample mass (0.0863 g) and the 5 mL digest volume. They were used only as benchmark exclusions in lower-tail sensitivity analyses and not as reconstructed sample-specific LOD or LOQ values.

#### 2.4.2. Secondary Mass-Adjusted Colony Cd Estimates and Variance Partitioning

Because dry sample mass also enters the denominator of dry-weight concentration, the mass-covariate model was treated as a secondary sensitivity analysis rather than as a biological correction. ln(Cd) was modeled with colony as a categorical predictor and centered ln(dry mass) as a covariate. Heteroscedasticity-robust standard errors (HC3) were used for inference on fixed effects. Back-transformed model estimates at the mean dry mass are reported as adjusted geometric means with 95% confidence intervals [29].

For descriptive variance partitioning, a random-intercept mixed model was fitted by maximum likelihood to ln(Cd), with centered ln(dry mass) retained as a fixed-effect covariate [29]. Variance partitioning was summarized using the intraclass correlation coefficient (ICC), defined as the ratio of between-colony variance to total variance [30]. A Wald confidence interval for the ratio of between-colony to residual variance was calculated on the logarithmic scale using the delta method and back-transformed, then converted to ICC values as r/(1 + r). Given only nine colonies, the ICC is treated as a model-dependent descriptive estimate rather than a precisely estimated biological fraction.

#### 2.4.3. Ecological and Spatial Structuring of Colony Cd

**Figure 4:**
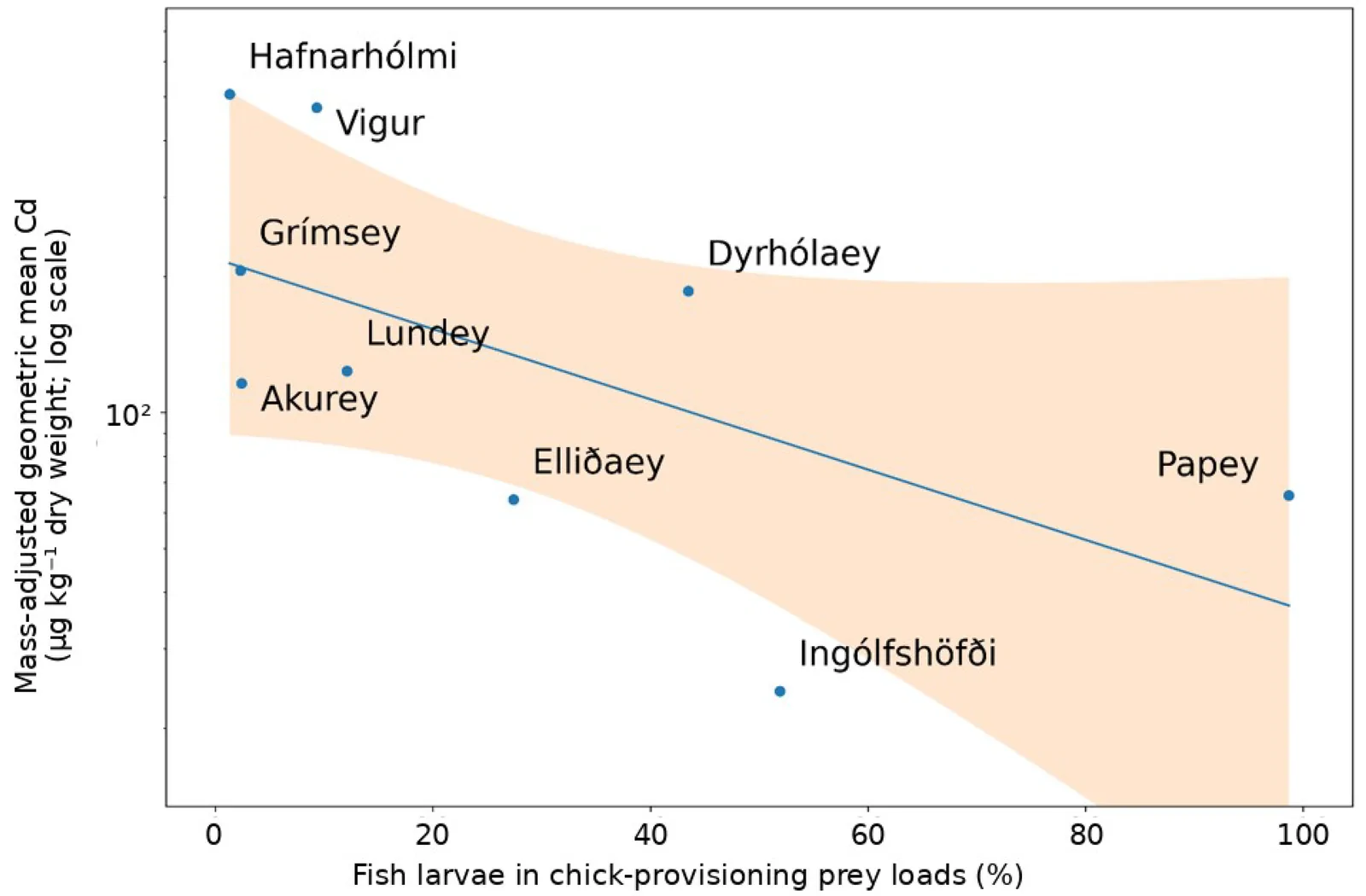
Colony-level cadmium concentration in fresh Atlantic puffin *Fratercula arctica* droppings and the proportion of fish larvae in chick-provisioning prey loads across nine Icelandic colonies, June–July 2018. Blue points show adjusted geometric means. The solid blue line shows the ordinary least-squares fit, and the orange shaded band represents its back-transformed 95% confidence interval. This was the strongest exploratory association (Spearman ρ = −0.733, *p* = 0.025, FDR-adjusted q = 0.196) and was not significant after false-discovery-rate correction. The fitted line and confidence band are included for descriptive visualization only.

Ecological structuring was evaluated at the colony level (*n* = 9) using mass-adjusted colony ln(Cd) estimates. Monotonic associations between adjusted ln(Cd) and colony-level diet proportions and breeding-performance metrics were assessed using Spearman rank correlations [31]. Productivity was treated as a derived index because it was calculated as burrow occupancy × nesting success rather than from a separate binary outcome count. To control the false discovery rate across the eight exploratory ecological screening tests (four prey-category correlations and four breeding-performance correlations), *p* values were adjusted using the Benjamini–Hochberg procedure [25], with results reported as q values. Because the four prey proportions sum to 100%, these category-specific correlations were treated as exploratory compositional summaries rather than independent mechanistic effects. Heterogeneity among colonies in prey-category counts and in binary breeding outcomes was evaluated using contingency-table chi-square tests, and effect size was reported as Cramér’s V. Wilson 95% confidence intervals were used for prey-category proportions. For burrow occupancy, hatching success, and nesting success, 95% confidence intervals were calculated as *p* ± 1.96 SE and truncated to the interval 0–1. The standard error of productivity was approximated by first-order propagation of uncertainty as SE(P) = P × √{[SE(NS)/NS]^2^ + [SE(BO)/BO]^2^}. For Figure 4 only, an ordinary least-squares model was fitted to mass-adjusted colony ln(Cd) as a function of the percentage of fish larvae. The fitted mean and its 95% confidence interval were back-transformed for visualization. Inferential screening of this relationship remained based on Spearman correlation with false-discovery-rate correction. The intervals for burrow occupancy, hatching success, and nesting success and the propagated uncertainty for productivity are treated as descriptive approximations and are not used as inferential inputs in the Cd association tests.

Longitude and latitude were tested as predictors of adjusted ln(Cd) using Spearman correlations [31] and simple ordinary least-squares regressions [32], based on the coordinates stated in Section 2.1. Global spatial autocorrelation was assessed using Moran’s I [22] with row-standardized inverse-distance weights derived from great-circle distances among colonies [23]. Significance was evaluated using 10,000 random permutations [33], with an add-one correction and a two-sided statistic based on |Iperm| ≥ |Iobserved|. The random-number seed was 2026.

## 3. Results

### 3.1. Cd Distributions in Droppings and Unadjusted Among-Colony Contrasts

**Figure 2:**
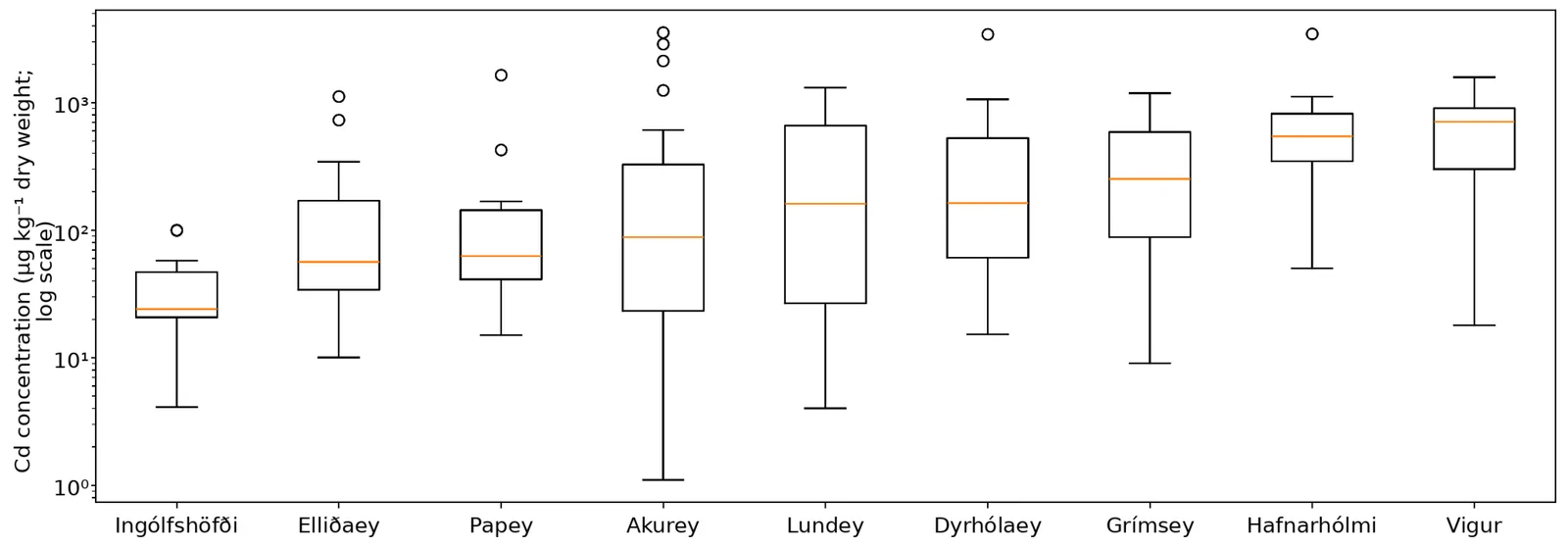
Cadmium concentrations in fresh droppings from Atlantic puffins *Fratercula arctica* across Icelandic colonies. Boxes indicate the interquartile range, the horizontal line indicates the median, whiskers extend to 1.5 times the interquartile range, and points denote observations identified as outliers under this convention. The number of points therefore varies among colonies according to the number of observations falling outside the 1.5 × IQR limits. Sample sizes are Ingólfshöfði *n* = 17, Elliðaey *n* = 14, Papey *n* = 14, Akurey *n* = 27, Lundey *n* = 15, Dyrhólaey *n* = 16, Grímsey *n* = 21, Hafnarhólmi *n* = 16, and Vigur *n* = 19. Concentrations are expressed as µg kg^−1^ dry weight and shown on a logarithmic scale.

Cadmium concentration and dry mass were non-normally distributed (Shapiro–Wilk, both *p* < 0.001), and Cd remained non-normal after log_10_ transformation (W = 0.975, *p* = 0.005). Cadmium concentrations in droppings ranged from 1.1 to 3539.2 µg kg^−1^ dry weight, with a median of 155.2 µg kg^−1^ and an interquartile range of 36.6–614.5 µg kg^−1^ (Figure 2). Dry sample mass ranged from 0.0006 to 0.0863 g (median, 0.0081 g; interquartile range, 0.0035–0.0165 g). Given the strong skewness, colony contrasts were summarized using medians and interquartile ranges alongside complementary log-scale modeling.

Marked colony-scale heterogeneity was observed in unadjusted Cd distributions in droppings (Figure 1 and Figure 2). The highest unadjusted medians were recorded at Vigur (706.6 µg kg^−1^ dry weight) and Hafnarhólmi (543.0 µg kg^−1^), whereas the lowest median occurred at Ingólfshöfði (24.0 µg kg^−1^). Consistent with these contrasts, a non-parametric omnibus test supported among-colony differences in Cd on the original scale (Kruskal–Wallis, H(8) = 45.36, *p* < 0.001). The among-colony effect remained significant after excluding reported concentrations < 11.6 µg kg^−1^ (*n* = 149; H(8) = 41.68, *p* = 1.56 × 10^−6^) and after excluding concentrations < 38.8 µg kg^−1^ (*n* = 118; H(8) = 34.41, *p* = 3.42 × 10^−5^). All nine colonies remained represented in both analyses. These benchmark exclusions assess lower-tail robustness only and do not establish sample-specific detection or quantification status.

Following the overall among-colony heterogeneity in Cd concentrations in droppings, Dunn’s post hoc tests with Holm adjustment identified 9 of 36 colony pairs with significant differences (adjusted *p* < 0.05). Vigur differed from Ingólfshöfði, Akurey, Elliðaey, and Papey (all adjusted *p* ≤ 0.033), while Hafnarhólmi differed from Ingólfshöfði, Akurey, Elliðaey, and Papey (adjusted *p* < 0.001–0.045). Grímsey also differed from Ingólfshöfði (adjusted *p* = 0.006). No other pairwise contrast remained statistically detectable after Holm correction. Complete results for all 36 pairwise comparisons are provided in Table S3.

### 3.2. Mass-Adjusted Colony Cd Estimates and Variance Partitioning

In the secondary mass-covariate analysis, the colony effect remained strong (Wald χ^2^(8) = 108.75, *p* < 0.001). Dry mass showed a small negative association with ln(Cd) (β = −0.269, *p* = 0.045), but this coefficient is not interpreted biologically because dry mass also enters the concentration denominator. Adjusted geometric means, back-transformed at mean dry mass, ranged from 24.2 µg kg^−1^ at Ingólfshöfði (95% confidence interval, 14.7–39.9) to 506.9 µg kg^−1^ at Hafnarhólmi (95% confidence interval, 320.3–802.2), a 20.9-fold model-based contrast.

The secondary random-intercept mixed model estimated that 26.1% of total ln(Cd) variance was attributable to between-colony differences (ICC = 0.261; 95% confidence interval, 0.104–0.519), whereas 73.9% occurred within colonies. On the natural-log scale, the between-colony variance component was approximately 0.35 times the within-colony residual variance, and the effect of centered ln(dry mass) remained negative (β = −0.249, *p* = 0.026). Given nine colonies and the wide confidence interval, this ICC is interpreted as a model-dependent descriptive estimate.

### 3.3. Diet Composition and Breeding Performance Among Colonies

**Figure 3:**
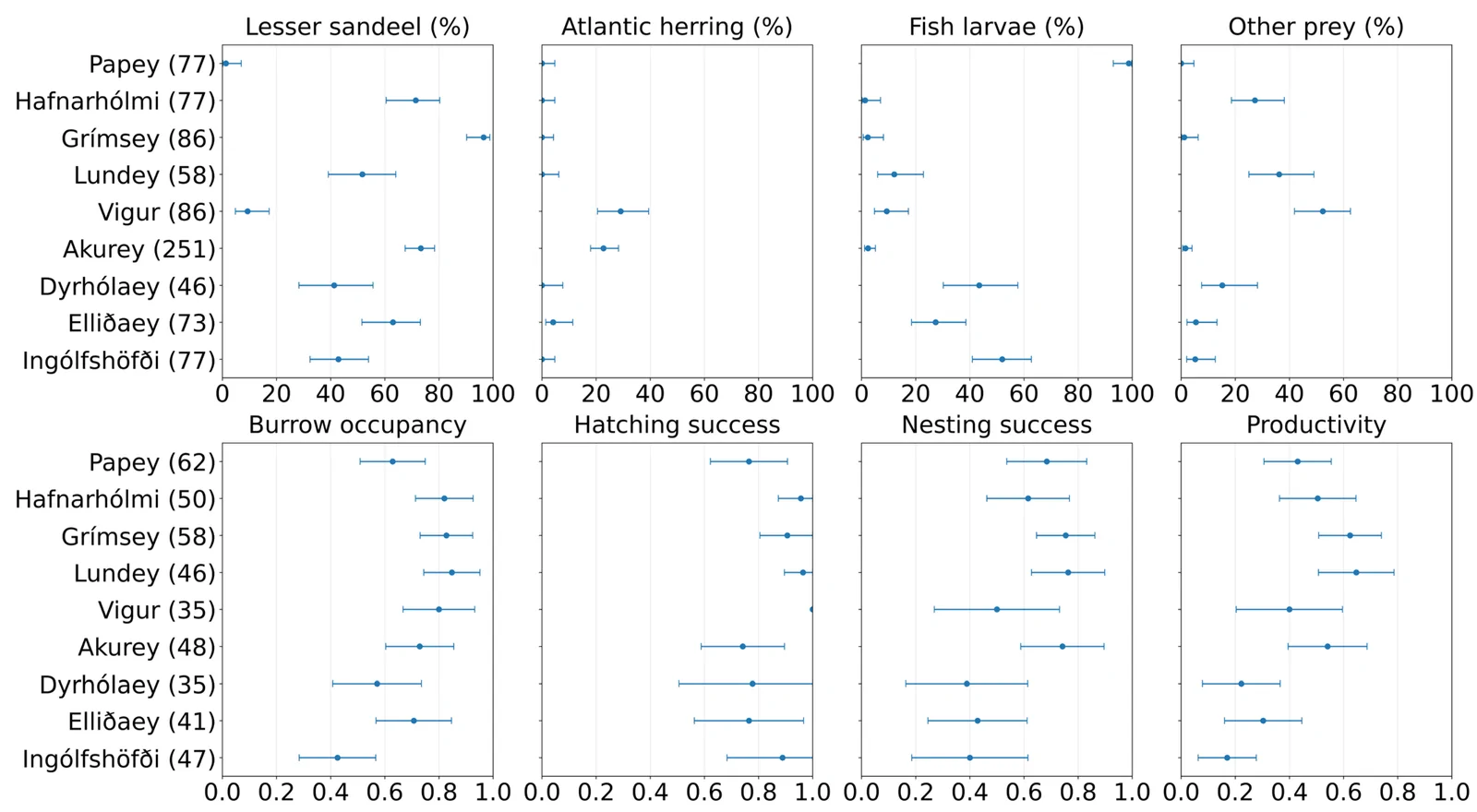
Diet composition (**top** panels; *n* in parentheses = analyzed photographs) and reproductive performance (**bottom** panels; *n* in parentheses = inspected burrows) in Icelandic Atlantic puffin *Fratercula arctica* colonies. Error bars show Wilson 95% confidence intervals for prey proportions, *p* ± 1.96 SE intervals truncated to 0–1 for burrow occupancy, hatching success, and nesting success, and approximate propagated 95% confidence intervals for productivity [16]. The numerical values underlying the panels are provided in Table S2. The breeding-performance intervals are descriptive approximations because complete event-level numerator and denominator records are not retained for all colony-by-outcome combinations.

Chick-provisioning prey composition differed strongly among colonies, based on 831 analyzed photographs of prey loads (Figure 3). A chi-square test supported heterogeneity in prey-category frequencies (χ^2^(24) = 764.4, *p* < 0.001), with a large effect size (Cramér’s V = 0.554). Decomposition of the Pearson χ^2^ statistic showed that fish larvae made the largest contribution to the among-colony diet heterogeneity (43.9% of total χ^2^), followed by “other” prey (25.1%), lesser sandeel (16.1%), and Atlantic herring (14.9%). Papey was particularly distinctive, with fish larvae recorded in 76 of 77 prey-load photographs (98.7%). These data describe prey delivered to chicks, not complete adult diet.

Breeding performance also varied among colonies (*n* = 422 monitored burrows). Heterogeneity was supported for burrow occupancy (χ^2^(8) = 36.14, *p* < 0.001; V = 0.293) and nesting success (χ^2^(8) = 24.36, *p* = 0.002; V = 0.289), whereas evidence for among-colony differences in hatching success was weaker (χ^2^(8) = 14.04, *p* = 0.081; V = 0.270). Observed ranges were 0.426–0.848 for burrow occupancy, 0.742–1.000 for hatching success, 0.389–0.763 for nesting success, and 0.170–0.647 for productivity (Figure 3).

### 3.4. Associations of Colony Cd with Diet, Breeding Performance, and Spatial Structure

Exploratory monotonic associations between adjusted colony ln(Cd) and colony-level predictors were evaluated using Spearman correlations (*n* = 9 colonies). The strongest association was a negative correlation with the proportion of fish larvae in the diet (ρ = −0.733, *p* = 0.025), but this association did not remain significant after correction for multiple comparisons (q = 0.196; Figure 4). No correlation reached the false-discovery-rate threshold (all q ≥ 0.196).

Across colonies (*n* = 9), no breeding-performance metric showed a negative monotonic association with mass-adjusted colony ln(Cd), and none remained detectable after false-discovery-rate correction: burrow occupancy, ρ = 0.617, *p* = 0.077, q = 0.205; hatching success, ρ = 0.628, *p* = 0.070, q = 0.205; nesting success, ρ = 0.217, *p* = 0.576, q = 0.728; and productivity, ρ = 0.400, *p* = 0.286, q = 0.458. Complete results for all eight exploratory colony-level associations, including exact unadjusted *p* values and Benjamini–Hochberg-adjusted q values, are provided in Table S2.

With only nine sites, no broad spatial autocorrelation was detectable in adjusted colony ln(Cd) (Moran’s I = −0.177, permutation *p* = 0.335). Longitude was not associated with ln(Cd) (Spearman ρ = 0.000, *p* = 1.000; ordinary least-squares slope = −0.019, *p* = 0.863), and latitude was not associated with ln(Cd) (Spearman ρ = 0.500, *p* = 0.170; ordinary least-squares slope = 0.452, *p* = 0.157). These negative spatial tests are interpreted cautiously because power is low at *n* = 9 colonies.

## 4. Discussion

This study tested whether Cd concentrations in droppings during chick rearing showed colony-scale heterogeneity across Iceland, ecological structuring linked to chick-provisioning prey composition, and an adverse association with breeding performance. The primary, unadjusted rank-based analysis supported strong among-colony heterogeneity. A secondary model including dry sample mass produced the same qualitative conclusion, with adjusted geometric means spanning more than 20-fold. In contrast, exploratory diet and breeding-performance associations were not supported after correction for multiple comparisons. The reported data therefore show a geographically fine-grained colony signal, while the limited number of colonies and absence of direct prey Cd measurements constrain inference about ecological drivers and demographic consequences.

Seabirds have been widely used as sentinels of environmental change and contamination because they integrate ecosystem processes across trophic levels and broad spatial scales, particularly during breeding when foraging is constrained around colonies [1,2]. In this context, the large among-colony contrasts observed in the primary unadjusted analysis and retained in the secondary mass-adjusted model demonstrate spatial heterogeneity in Cd concentrations in droppings. Such heterogeneity may be consistent with differences in recent dietary intake within colony-specific foraging areas, but the present data do not identify a specific exposure pathway. Atlantic puffins are central-place foragers during chick rearing, and breeding outcomes and foraging costs respond strongly to prey-field variation and prey shortages [13,34]. The magnitude of the colony effect may therefore reflect colony-level differences in prey availability or prey Cd burden within commuting range, among other local processes, although direct prey sampling is required to attribute differences to specific pathways [5,13].

Variance partitioning further indicated that colony membership was informative but not determinative. The secondary model estimated that approximately 26% of total ln(Cd) variance was attributable to between-colony differences, with most variance occurring within colonies. This estimate is model-dependent and uncertain with only nine colonies (95% confidence interval, 0.104–0.519). High within-colony variability is biologically plausible because puffins from the same colony can differ in space use, foraging, and prey choice [35,36]. From a monitoring perspective, the combination of a colony signal and high within-colony dispersion implies that adequate within-colony replication is important [4,10].

The negative association between colony ln(Cd) and the proportion of fish larvae in chick-provisioning prey-load photographs did not remain detectable after false-discovery-rate correction and should be treated as hypothesis-generating rather than confirmatory. One possible explanation is that larval fish carried to chicks differed in Cd content from other prey categories. However, Cd was not measured directly in prey, prey-load photographs do not represent the complete adult diet of the birds producing the droppings, and the prey proportions are compositional. Because consistent biomagnification of Cd has not been observed in aquatic food webs, differences among prey taxa, life stages, local exposure histories, and individual foraging strategies may be more important than trophic position alone [37,38].

Spatial and temporal variation in Cd concentrations in fish, including in Icelandic waters, has been documented, and variation in prey Cd among colonies may therefore be obscured by broad prey categories [39]. The present data cannot distinguish whether the observed pattern reflects prey composition, colony-specific Cd concentrations within prey taxa, or environmental Cd availability. Inference is further limited by the small number of colonies, coarse prey categorization, and known method-specific biases of chick-diet proxies based on visible prey loads. Reviews and comparative evaluations have emphasized that dietary methods capture different components of diet and differ in taxonomic resolution and representativeness [14,40]. A prey-related Cd signal may therefore have been attenuated by measurement error and aggregation at the colony level rather than being absent.

No evidence was obtained for the hypothesized negative association between colony-level Cd and breeding performance. This should not be interpreted as evidence that cadmium is irrelevant to Atlantic puffin demography. First, screening was conducted at the colony level (*n* = 9), limiting power unless relationships were strong and monotonic. Second, breeding performance in puffins is shaped by multiple high-variance drivers, particularly prey availability and foraging conditions, whose effects can dominate inter-colony contrasts in a given season [13,34]. Third, Cd concentrations in droppings may be influenced by relatively recent dietary intake and do not provide a direct measure of internal accumulation or chronic body burden.

Methodological syntheses and empirical evaluations have emphasized that droppings can be informative bioindicators, but their mixed fecal and urate composition, physiological processes, and element-specific kinetics complicate the relationship between excreted concentrations and internal stores [4,10]. Colony differences may therefore reflect multiple interacting processes rather than prey contamination alone, including sex- or individual-specific foraging, temporal changes in prey availability during June–July, digestive or metabolic variation, renal excretion, hydration, and fecal–urate composition. Consequently, the absence of a colony-level relationship between Cd in droppings and breeding indices is compatible with several scenarios and should not be interpreted as evidence about internal Cd body burden.

Spatial analyses showed no detectable spatial autocorrelation and no longitude or latitude effect, indicating that Cd heterogeneity was not described by a simple regional gradient. Instead, the results imply a patchy landscape of Cd concentrations in droppings, potentially reflecting localized oceanographic, trophic, or geogenic processes that generate strong colony contrasts without producing broad spatial structuring detectable at nine sites. Iceland is characterized by pronounced spatiotemporal variability in dust emission and atmospheric transport, which may contribute to spatial heterogeneity in mineral-associated deposition at land–sea interfaces [15]. The contribution of such processes to cadmium availability in puffin prey cannot be resolved from the present data, and multiple local drivers may shape the observed Cd pattern in droppings during chick rearing [15,36].

Interpretation across biomonitoring matrices also warrants caution. Among-colony differences in metals, including Cd, have been reported in Atlantic puffin feathers from Iceland [11]. However, feathers integrate exposure during feather growth and are influenced by molt timing and potentially by external deposition, whereas droppings may be more strongly influenced by recent dietary intake and excretory physiology [4,10]. Feathers therefore require cautious interpretation as indicators of internal accumulation [41].

Metal patterns in Atlantic puffin tissues and feathers have also been documented outside Iceland, supporting the broader inference that matrix choice determines the temporal window and biological meaning of exposure estimates [42]. Concordance in colony rank order between feathers and droppings should not be assumed. Mismatches between matrices may instead provide information about different exposure windows and pathways [4,10,11].

The principal contribution of this study is the demonstration that reported Cd concentrations in non-invasively collected fresh droppings showed strong colony-scale structure across Iceland in the 2018 breeding season. The analytical sensitivity values available for this series are solution-based and are not directly transferable to sample-specific dry-weight thresholds because the latter depend on the amount of material represented in each digest and the corresponding sample calculation. The original sample-level analytical records required to assign dry-weight detection and quantification status are not retained, and repeat determination is not possible because no residual digests or sample material remain. Accordingly, the analytical status of the lowest individual observations remains unresolved. Importantly, the primary rank-based colony effect persisted after excluding reported concentrations below 11.6 and 38.8 µg kg^−1^ in the two benchmark analyses, showing that the overall among-colony heterogeneity is not attributable solely to the lowest observations under those benchmark cut-offs. These sensitivity analyses do not validate any individual low-end measurement and do not substitute for sample-specific method limits. Because no field blanks were collected, incidental field contamination cannot be independently quantified. Published work on avian droppings likewise shows that interpretation depends strongly on species, contamination setting, and fecal–urate composition [4,10], limiting direct quantitative comparison among studies. Future studies should combine concurrent sampling of representative prey, higher-resolution diet tools such as DNA metabarcoding, multi-year replication, and preservation of complete sample-level analytical metadata to distinguish persistent spatial patterns from interannual variability [13,14,34].

## 5. Conclusions

Reported cadmium concentrations in fresh droppings from Atlantic puffins differed strongly among nine Icelandic colonies in the primary unadjusted rank-based analysis (Kruskal–Wallis, *p* < 0.001; 9 of 36 Holm-adjusted Dunn comparisons significant). The primary colony contrast also remained significant in both lower-tail benchmark-exclusion analyses (<11.6 µg kg^−1^: *n* = 149, H(8) = 41.68, *p* < 0.001; < 38.8 µg kg^−1^: *n* = 118, H(8) = 34.41, *p* < 0.001). A secondary mass-covariate model yielded adjusted geometric means spanning 20.9-fold, and a secondary mixed model yielded an ICC of 0.261 (95% confidence interval, 0.104–0.519); these model-based quantities are interpreted cautiously. The chick-provisioning prey-load data did not explain the colony pattern after multiplicity correction and should not be interpreted as the complete adult diet. No broad spatial gradient was detectable with nine sites.

Fresh-dropping biomonitoring can therefore complement existing seabird monitoring by identifying spatial contrasts without capturing or handling birds. Because sample-specific dry-weight analytical limits cannot be assigned from the retained historical records, the lowest reported concentrations should be treated as analytically unresolved rather than as verified quantitative measurements, and they should not be used to infer internal body burden. The benchmark-exclusion analyses support the robustness of the overall colony contrast but do not retrospectively validate individual low-end observations. The central inference from this historical 2018 dataset is therefore comparative: reported Cd concentrations in droppings showed strong colony-scale structure. Adequate within-colony replication, standardized sample processing, complete analytical archiving, direct measurements of Cd in prey, and repeated sampling across years are necessary before the observed differences can be attributed to specific sources or demographic effects.

## Data Availability

Individual reported Cd concentrations are provided in Table S1, the colony-level data underlying Figure 3 together with complete results of the eight exploratory ecological associations are provided in Table S2, and complete Dunn pairwise results are provided in Table S3. The 2018 chick-provisioning and breeding-monitoring information is available in the cited national monitoring report [16]. The retained analytical archive contains the final reported dry-weight Cd concentrations and the general method and quality-control information described in Section 2.2 and Section 2.3. The retained breeding dataset contains the colony-level summaries in Table S2 but not complete event-level numerator and denominator records for all colony-by-outcome combinations.

## Supplementary Materials

The following supporting information can be downloaded at https://www.mdpi.com/article/10.3390/ani16172689/s1: Table S1. Individual reported cadmium (Cd) concentrations (μg kg^−1^ dry weight) in individual droppings of Atlantic puffin *Fratercula arctica* collected across nine Icelandic colonies during the breeding season (June-July) 2018; Table S2. Colony-level chick-provisioning prey composition and breeding-performance data underlying Figure 3, and complete results of the eight exploratory associations with mass-adjusted colony ln(Cd); Table S3. Complete Dunn pairwise comparisons of reported cadmium (Cd) concentrations in Atlantic puffin *Fratercula arctica* droppings among nine Icelandic colonies, with Holm-adjusted *p* values.
