# Supplementary material for: Non-Invasive Sampling Reveals Colony-Scale Spatial Heterogeneity in Cadmium Concentrations in Atlantic Puffin Fratercula arctica Droppings in Iceland
Source: Animals (Basel). 2026 Aug 28;16(17):2689. doi: 10.3390/ani16172689 (PMC13564283; doi:10.3390/ani16172689)
Supplement: Supplementary file 1 [file animals-16-02689-s001.zip › animals-4483927-supplementary.pdf]

Table S1. Individual reported cadmium (Cd) concentrations (µg kg^{-1} dry weight) in individual droppings of Atlantic puffin *Fratercula arctica* collected across nine Icelandic colonies during the breeding season (June-July) 2018

**Sample ID**	**Population**	**Cd**	**Sample ID**	**Population**	**Cd**	**Sample ID**	**Population**	**Cd**	**Sample ID**	**Population**	**Cd**	**Sample ID**	**Population**	**Cd**
A1	Akurey	34.8	G1	Grímsey	449.0	V1	Vigur	706.6	D1	Dyrhólaey	478.9	I1	Ingólfshöfði	100.2
A2	Akurey	84.5	G2	Grímsey	404.0	V2	Vigur	735.2	D2	Dyrhólaey	1059.0	I2	Ingólfshöfði	99.3
A3	Akurey	123.6	G3	Grímsey	162.0	V3	Vigur	818.3	D3	Dyrhólaey	15.2	I3	Ingólfshöfði	34.0
A4	Akurey	10.8	G4	Grímsey	801.0	V4	Vigur	653.1	D4	Dyrhólaey	23.8	I4	Ingólfshöfði	24.0
A5	Akurey	222.7	G5	Grímsey	1182.0	V5	Vigur	853.5	D5	Dyrhólaey	68.8	I5	Ingólfshöfði	10.1
A6	Akurey	88.0	G6	Grímsey	587.0	V6	Vigur	348.2	D6	Dyrhólaey	275.3	I6	Ingólfshöfði	12.1
A7	Akurey	1.1	G7	Grímsey	88.0	V7	Vigur	253.1	D7	Dyrhólaey	663.2	I7	Ingólfshöfði	42.9
A8	Akurey	24.4	G8	Grímsey	37.0	V8	Vigur	17.9	D8	Dyrhólaey	127.7	I8	Ingólfshöfði	21.1
A9	Akurey	149.8	G9	Grímsey	354.0	V9	Vigur	923.9	D9	Dyrhólaey	143.2	I9	Ingólfshöfði	7.6
A10	Akurey	366.6	G10	Grímsey	66.0	V10	Vigur	94.9	D10	Dyrhólaey	198.8	I10	Ingólfshöfði	21.5
A11	Akurey	19.6	G11	Grímsey	43.0	V11	Vigur	186.8	D11	Dyrhólaey	930.1	I11	Ingólfshöfði	4.1
A12	Akurey	285.0	G12	Grímsey	746.0	V12	Vigur	1145.0	D12	Dyrhólaey	3426.0	I12	Ingólfshöfði	46.7
A13	Akurey	608.9	G13	Grímsey	851.0	V13	Vigur	1110.0	D13	Dyrhólaey	89.5	I13	Ingólfshöfði	22.5
A14	Akurey	28.2	G14	Grímsey	191.0	V14	Vigur	876.8	D14	Dyrhólaey	19.2	I14	Ingólfshöfði	47.9
A15	Akurey	28.7	G15	Grímsey	251.0	V15	Vigur	598.1	D15	Dyrhólaey	181.1	I15	Ingólfshöfði	39.1
A16	Akurey	21.9	G16	Grímsey	769.0	V16	Vigur	1580.0	D16	Dyrhólaey	36.1	I16	Ingólfshöfði	20.7
A17	Akurey	241.6	G17	Grímsey	538.0	V17	Vigur	456.3				I17	Ingólfshöfði	57.7
A18	Akurey	6.9	G18	Grímsey	122.0	V18	Vigur	229.0						
A19	Akurey	13.1	G19	Grímsey	252.0	V19	Vigur	1478.0						
A20	Akurey	18.9	G20	Grímsey	18.0									
A21	Akurey	49.0	G21	Grímsey	9.0									
A22	Akurey	1247.9												
A23	Akurey	384.5												
A24	Akurey	2119.8												
A25	Akurey	133.5												
A26	Akurey	2875.5												
A27	Akurey	3539.2												

Sample ID	Population	Cd	Sample ID	Population	Cd	Sample ID	Population	Cd	Sample ID	Population	Cd
H1	Hafnarhólmi	281.0	L1	Lundey	24.3	P1	Papey	106.3	E1	Elliðaey	188.7
H2	Hafnarhólmi	865.0	L2	Lundey	491.2	P2	Papey	44.2	E2	Elliðaey	1114.8
H3	Hafnarhólmi	3461.0	L3	Lundey	680.5	P3	Papey	15.0	E3	Elliðaey	53.3
H4	Hafnarhólmi	439.0	L4	Lundey	29.0	P4	Papey	78.8	E4	Elliðaey	52.9
H5	Hafnarhólmi	801.0	L5	Lundey	634.5	P5	Papey	1643.1	E5	Elliðaey	41.8
H6	Hafnarhólmi	50.0	L6	Lundey	160.7	P6	Papey	155.2	E6	Elliðaey	112.7
H7	Hafnarhólmi	673.0	L7	Lundey	679.7	P7	Papey	44.0	E7	Elliðaey	59.1
H8	Hafnarhólmi	1110.0	L8	Lundey	1304.4	P8	Papey	25.0	E8	Elliðaey	31.4
H9	Hafnarhólmi	313.0	L9	Lundey	77.7	P9	Papey	46.3	E9	Elliðaey	77.5
H10	Hafnarhólmi	358.0	L10	Lundey	1240.0	P10	Papey	40.0	E10	Elliðaey	10.0
H11	Hafnarhólmi	396.0	L11	Lundey	6.1	P11	Papey	28.0	E11	Elliðaey	15.8
H12	Hafnarhólmi	620.0	L12	Lundey	13.8	P12	Papey	424.8	E12	Elliðaey	728.0
H13	Hafnarhólmi	1031.0	L13	Lundey	144.9	P13	Papey	86.0	E13	Elliðaey	15.5
H14	Hafnarhólmi	466.0	L14	Lundey	278.1	P14	Papey	167.4	E14	Elliðaey	343.2
H15	Hafnarhólmi	656.0	L15	Lundey	4.0						
H16	Hafnarhólmi	282.0									

Table S2. Colony-level chick-provisioning prey composition and breeding-performance data underlying Figure 3, and complete results of the eight exploratory associations with mass-adjusted colony ln(Cd)

A. Colony-level numerical data underlying Figure 3

Colony	N_B	BO ± SE	HS ± SE	NS ± SE	P ± SE	N_D	Lesser sandeel (%) ± SE	Atlantic herring (%) ± SE	Fish larvae (%) ± SE	Other prey (%) ± SE
Papey	62	0.6290 ± 0.0613	0.7647 ± 0.0727	0.6842 ± 0.0754	0.4304 ± 0.1472	77	1.30 ± 1.29	0.00 ± 0.00	98.70 ± 1.29	0.00 ± 0.00
Hafnarhólmi	50	0.8200 ± 0.0543	0.9565 ± 0.0425	0.6154 ± 0.0779	0.5046 ± 0.1429	77	71.40 ± 5.15	0.00 ± 0.00	1.30 ± 1.29	27.30 ± 5.08
Grímsey	58	0.8276 ± 0.0496	0.9062 ± 0.0515	0.7541 ± 0.0551	0.6241 ± 0.0945	86	96.50 ± 1.98	0.00 ± 0.00	2.30 ± 1.63	1.20 ± 1.16
Lundey	46	0.8478 ± 0.0530	0.9643 ± 0.0351	0.7632 ± 0.0690	0.6470 ± 0.1099	58	51.70 ± 6.56	0.00 ± 0.00	12.10 ± 4.28	36.20 ± 6.31
Vigur	35	0.8000 ± 0.0676	1.0000 ± 0.0000	0.5000 ± 0.1179	0.4000 ± 0.2504	86	9.30 ± 3.13	29.10 ± 4.90	9.30 ± 3.13	52.30 ± 5.39
Akurey	48	0.7292 ± 0.0641	0.7419 ± 0.0786	0.7419 ± 0.0786	0.5410 ± 0.1377	251	73.30 ± 2.79	22.70 ± 2.64	2.40 ± 0.96	1.60 ± 0.79
Dyrhólaey	35	0.5714 ± 0.0836	0.7778 ± 0.1386	0.3889 ± 0.1149	0.2222 ± 0.3297	46	41.30 ± 7.26	0.00 ± 0.00	43.50 ± 7.31	15.20 ± 5.30
Elliðaey	41	0.7073 ± 0.0711	0.7647 ± 0.1029	0.4286 ± 0.0935	0.3031 ± 0.2402	73	63.00 ± 5.65	4.10 ± 2.32	27.40 ± 5.22	5.50 ± 2.66
Ingólfshöfði	47	0.4255 ± 0.0721	0.8889 ± 0.1048	0.4000 ± 0.1095	0.1702 ± 0.3221	77	42.90 ± 5.64	0.00 ± 0.00	51.90 ± 5.69	5.20 ± 2.53

N_B = number of monitored burrows; BO = burrow occupancy; HS = hatching success; NS = nesting success; P = productivity; N_D = number of analyzed prey-load photographs; SE = standard error. Prey-load data describe chick provisioning. Colony-level ecological data are from the 2018 Icelandic puffin monitoring dataset (Hansen 2018), as used in the manuscript.

B. Complete eight-association exploratory screening results

Variable group	Predictor	Spearman ρ	p (unadjusted)	q (BH-FDR)	n	Missing
Chick-provisioning prey composition	Lesser sandeel proportion (%)	0.183333	0.636820	0.727794	9	0
Chick-provisioning prey composition	Atlantic herring proportion (%)	0.029704	0.939531	0.939531	9	0
Chick-provisioning prey composition	Fish larvae proportion (%)	-0.733333	0.024554	0.196433	9	0
Chick-provisioning prey composition	Other prey proportion (%)	0.483333	0.187470	0.374940	9	0
Breeding performance	Burrow occupancy	0.616667	0.076929	0.205143	9	0
Breeding performance	Hatching success	0.627621	0.070365	0.205143	9	0
Breeding performance	Nesting success	0.216667	0.575515	0.727794	9	0
Breeding performance	Productivity	0.400000	0.286105	0.457768	9	0

Associations are Spearman rank correlations between mass-adjusted colony ln(Cd) and four chick-provisioning prey-category proportions or four breeding-performance metrics. The eight tests form one multiplicity family; q values are Benjamini-Hochberg false-discovery-rate adjustments across all eight tests. All tests use n = 9 colonies and have no missing colony-level values. Because the four prey proportions sum to 100%, those category-specific correlations are exploratory compositional summaries rather than independent mechanistic effects.

Table S3. Complete Dunn pairwise comparisons of reported cadmium (Cd) concentrations in Atlantic puffin *Fratercula arctica* droppings among nine Icelandic colonies, with Holm-adjusted p values

Colony 1	Colony 2	Dunn z	p (unadjusted)	p (Holm)	Significant (Holm)
Ingólfshöfði	Elliðaey	-1.745174	0.080955	1.000000	No
Ingólfshöfði	Papey	-1.775263	0.075854	1.000000	No
Ingólfshöfði	Akurey	-2.482447	0.013048	0.326209	No
Ingólfshöfði	Lundey	-2.779366	0.005447	0.141609	No
Ingólfshöfði	Dyrhólaey	-3.055319	0.002248	0.060702	No
Ingólfshöfði	Grímsey	-3.756422	0.000172	0.005860	Yes
Ingólfshöfði	Hafnarhólmi	-5.151954	2.57786e-07	9.02251e-06	Yes
Ingólfshöfði	Vigur	-5.370491	7.85227e-08	2.82682e-06	Yes
Elliðaey	Papey	-0.028731	0.977079	1.000000	No
Elliðaey	Akurey	-0.421320	0.673521	1.000000	No
Elliðaey	Lundey	-0.954594	0.339783	1.000000	No
Elliðaey	Dyrhólaey	-1.186934	0.235254	1.000000	No
Elliðaey	Grímsey	-1.726537	0.084251	1.000000	No
Elliðaey	Hafnarhólmi	-3.182466	0.001460	0.042348	Yes
Elliðaey	Vigur	-3.302157	0.000959	0.030702	Yes
Papey	Akurey	-0.388347	0.697759	1.000000	No
Papey	Lundey	-0.925371	0.354773	1.000000	No
Papey	Dyrhólaey	-1.157260	0.247166	1.000000	No
Papey	Grímsey	-1.695064	0.090063	1.000000	No
Papey	Hafnarhólmi	-3.152792	0.001617	0.045281	Yes
Papey	Vigur	-3.271326	0.001070	0.033184	Yes
Akurey	Lundey	-0.670682	0.502423	1.000000	No
Akurey	Dyrhólaey	-0.936989	0.348764	1.000000	No
Akurey	Grímsey	-1.570519	0.116294	1.000000	No
Akurey	Hafnarhólmi	-3.251733	0.001147	0.034411	Yes
Akurey	Vigur	-3.420748	0.000624	0.020608	Yes
Lundey	Dyrhólaey	-0.221579	0.824641	1.000000	No
Lundey	Grímsey	-0.712810	0.475963	1.000000	No
Lundey	Hafnarhólmi	-2.253563	0.024224	0.557145	No
Lundey	Vigur	-2.340368	0.019265	0.462354	No
Dyrhólaey	Grímsey	-0.486191	0.626832	1.000000	No
Dyrhólaey	Hafnarhólmi	-2.065572	0.038869	0.816247	No
Dyrhólaey	Vigur	-2.147645	0.031742	0.698324	No
Grímsey	Hafnarhólmi	-1.714525	0.086432	1.000000	No
Grímsey	Vigur	-1.791968	0.073138	1.000000	No
Hafnarhólmi	Vigur	0.004632	0.996304	1.000000	No

Dunn's post hoc tests were applied following the Kruskal-Wallis comparison of the 159 reported Cd concentrations in Table S1. Holm adjustment was applied across all 36 pairwise comparisons. 'Yes' denotes Holm-adjusted $p < 0.05$.
